# Air bubbles around the heart: a rare case report of pneumohydropericardium in heart transplantation

**DOI:** 10.1093/ehjcr/ytaf062

**Published:** 2025-02-04

**Authors:** Filippo Brucato, Piero Gentile, Francesco Musca, Francesca Casadei, Matteo Palazzini

**Affiliations:** Department of Medicine and Surgery, University of Milano-Bicocca, Piazza dell'Ateneo Nuovo, 1, Milan 20126, Italy; Department of Cardiology, De Gasperis Cardio Center, ASST Grande Ospedale Metropolitano Niguarda, Piazza dell’Ospedale Maggiore, 3, Milan 20162, Italy; Department of Cardiology, De Gasperis Cardio Center, ASST Grande Ospedale Metropolitano Niguarda, Piazza dell’Ospedale Maggiore, 3, Milan 20162, Italy; Department of Cardiology, De Gasperis Cardio Center, ASST Grande Ospedale Metropolitano Niguarda, Piazza dell’Ospedale Maggiore, 3, Milan 20162, Italy; Department of Cardiology, De Gasperis Cardio Center, ASST Grande Ospedale Metropolitano Niguarda, Piazza dell’Ospedale Maggiore, 3, Milan 20162, Italy

**Keywords:** Pericardial effusion, Echocardiography, Cardiac transplant, Cardiomyopathy, Complication, Case report

## Abstract

**Background:**

Pneumohydropericardium is a rare but significant condition characterized by the presence of both air and fluid in the pericardial space. It is often associated with trauma, invasive procedures, or thoracic pathologies, and its occurrence following heart transplantation is particularly unusual. Early recognition is essential to prevent complications such as cardiac tamponade, although conservative management can be effective in cases without haemodynamic compromise.

**Case summary:**

We present the case of a 40-year-old woman with a history of hypokinetic-dilative cardiomyopathy and heterozygous for TNNT2 gene mutation (c.547C>T p.Arg183Trp), who underwent orthotopic heart transplantation. Following the procedure, she developed pneumohydropericardium, identified by echocardiography as air microbubbles and fluid in the pericardial space, without associated haemodynamic instability. The condition was monitored with serial echocardiograms, and no invasive intervention was required. The air microbubbles resolved after 16 days of echocardiographic follow-up, and the patient was discharged in stable condition. At follow-up, she showed no recurrence of the pneumohydropericardium.

**Discussion:**

This case highlights pneumohydropericardium as a rare post-transplant complication that, when detected early and closely monitored, can be managed conservatively without severe outcomes. While pneumohydropericardium has been previously reported in association with other conditions, its post-transplant manifestation is rare and warrants further awareness. Echocardiographic surveillance played a key role in guiding management, as invasive procedures were avoided, and the patient remained haemodynamically stable throughout. This case underscores the importance of recognizing and managing pneumohydropericardium in heart transplant patients to ensure optimal outcomes

**Learning points**

- Novelty: This case report addresses a rare post-transplant complication, pneumohydropericardium, making it a noteworthy contribution to medical literature, particularly in cardiovascular surgery and imaging.
- Educational value: The case emphasizes the utility of echocardiographic monitoring in avoiding invasive procedures and underscores the importance of conservative management in stable patients.

## Introduction

Pneumohydropericardium, characterized by the presence of both air and fluid in the pericardial space, is a rare but significant complication in cardiac care. Its aetiology varies, often associated with traumatic injury, invasive procedures, or underlying pathologies,^1–8^ while its occurrence following heart transplantation is rare. Early identification and management of this condition are crucial to prevent complications such as cardiac tamponade or haemodynamic instability.^9^ While the condition can be life-threatening in certain cases, it may also be managed conservatively without severe consequences if detected early.^9–12^ This case report illustrates a unique instance of pneumohydropericardium post-heart transplantation, emphasizing the importance of vigilant echocardiographic monitoring in guiding treatment. By documenting this rare case, we aim to contribute valuable insights into the post-transplant care of heart recipients and expand the current understanding of pneumohydropericardium's presentation and non-invasive management.

## Summary figure

**Figure:**
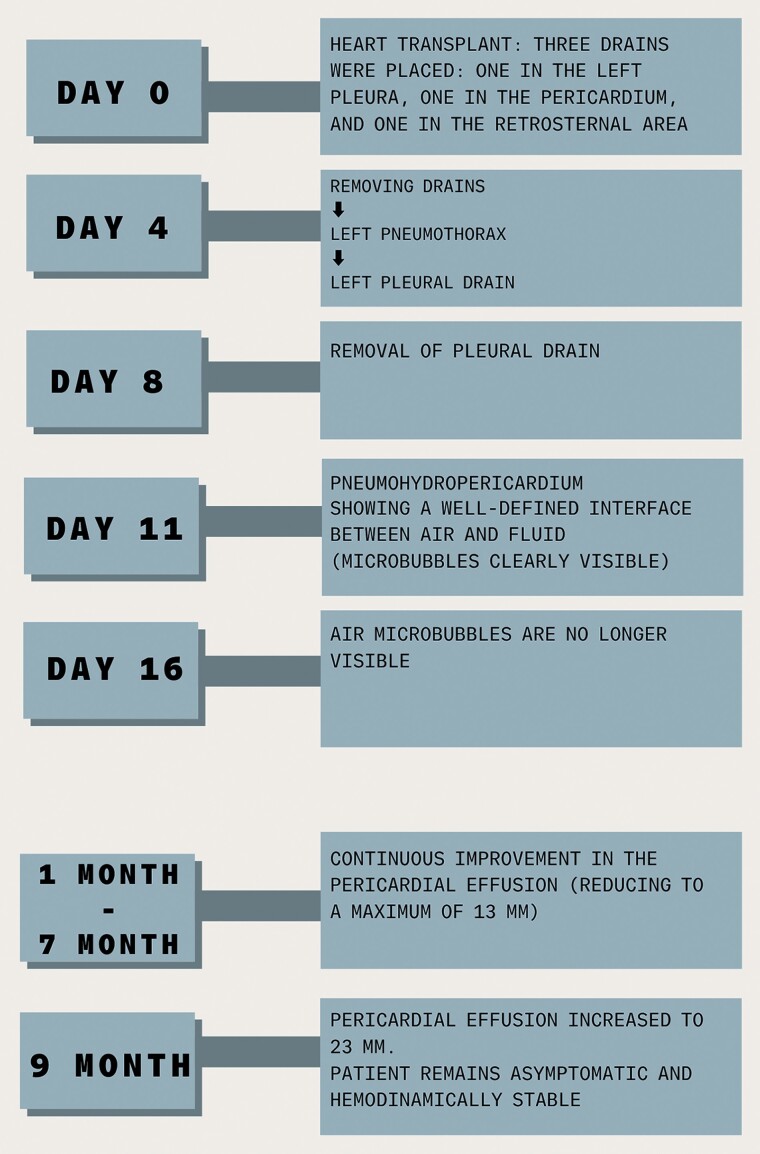


## Case presentation

A 40-year-old woman, heterozygous for a TNNT2 gene mutation (c.547C>T p.Arg183Trp), underwent orthotopic heart transplantation due to the development of hypokinetic-dilative cardiomyopathy complicated by heart failure and ventricular arrhythmias. The patient’s clinical history began in January 2017, when she started experiencing progressive dyspnoea and fatigue, with no history of syncope or presyncope. In February 2018, she suffered a cardiac arrest due to ventricular fibrillation at home during the 26th week of pregnancy. After a 10-min cardiopulmonary resuscitation by a layperson and subsequent intervention by emergency services, she was defibrillated and intubated. An emergency caesarean section was performed, complicated by haemorrhage, which required multiple transfusions. Coronary angiography showed no significant stenosis.

She was admitted to the intensive care unit from February 2018 and treated with intravenous inotropes (dobutamine), which were discontinued after about 24 h, and oral diuretics. Echocardiography revealed an enlarged left ventricle with severely reduced ejection fraction, moderate mitral insufficiency, and a dilated, hypokinetic right ventricle.

In March 2018, she was transferred to Niguarda Hospital, stable haemodynamically, with no evidence of hepatic or renal damage. A cardiac magnetic resonance imaging indicated severe left ventricular dilatation and dysfunction and moderate right ventricular dysfunction. Pre-discharge echocardiography showed slightly improved ejection fraction.

A subcutaneous implantable cardioverter-defibrillator (ICD) was implanted in March 2018 for secondary prevention. Subsequent echocardiographies showed stable dimensions and function of the ventricles, with a moderate improvement in mitral insufficiency. She was switched from enalapril to Entresto in May 2018, with regular cardiac check-ups thereafter.

She experienced an appropriate ICD intervention for polymorphic ventricular tachycardia in July 2021, with no apparent triggers. During a hospital stay, extensive ventricular ectopy was noted, and beta-blocker therapy was optimized, with the addition of mexiletine.

January 2022 saw her transferred for ventricular arrhythmia, with amiodarone introduced. A cardiac transplant was considered due to severe ventricular dysfunction and a history of ventricular arrhythmia.

Her condition remained stable until January 2024, when she developed a bronchitic episode but recovered without significant issues. However, persistent coughing continued.

In February 2024, she was admitted following a compatible donor alert, marking the initiation of the heart transplantation process.

Following the transplant, three drainages were placed: one in the left pleura, one in the pericardium, and one retrosternal.

On Day 4, the drains were removed, a procedure complicated by a left pneumothorax for which a left pleural drain was placed.

On Day 8, the pleural drainage was removed.

**Figure 1:**
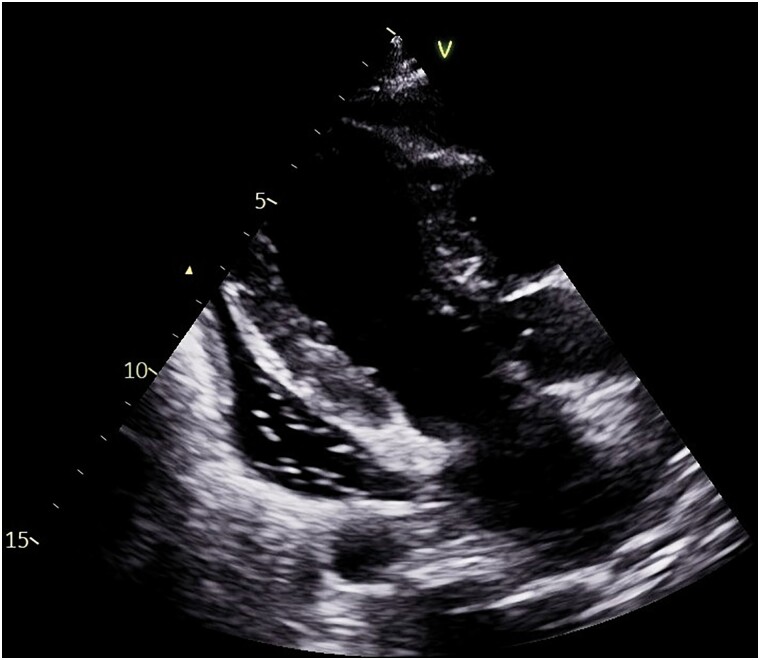
Parasternal long-axis findings in pneumohydropericardium. Parasternal long-axis view showing the moderate pericardial effusion (21 mm) with clear visualization of air bubbles posterior to the left ventricle.

**Figure 2:**
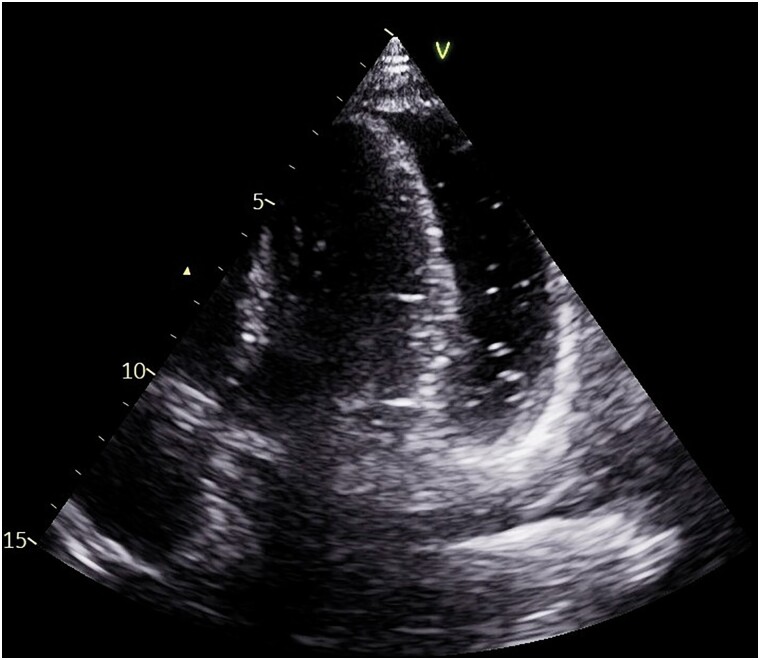
Apical four-chamber findings in pneumohydropericardium. Apical four-chamber view showing moderate pericardial effusion with clear visualization of air bubbles in front of the left ventricular anterolateral wall.

**Figure 3:**
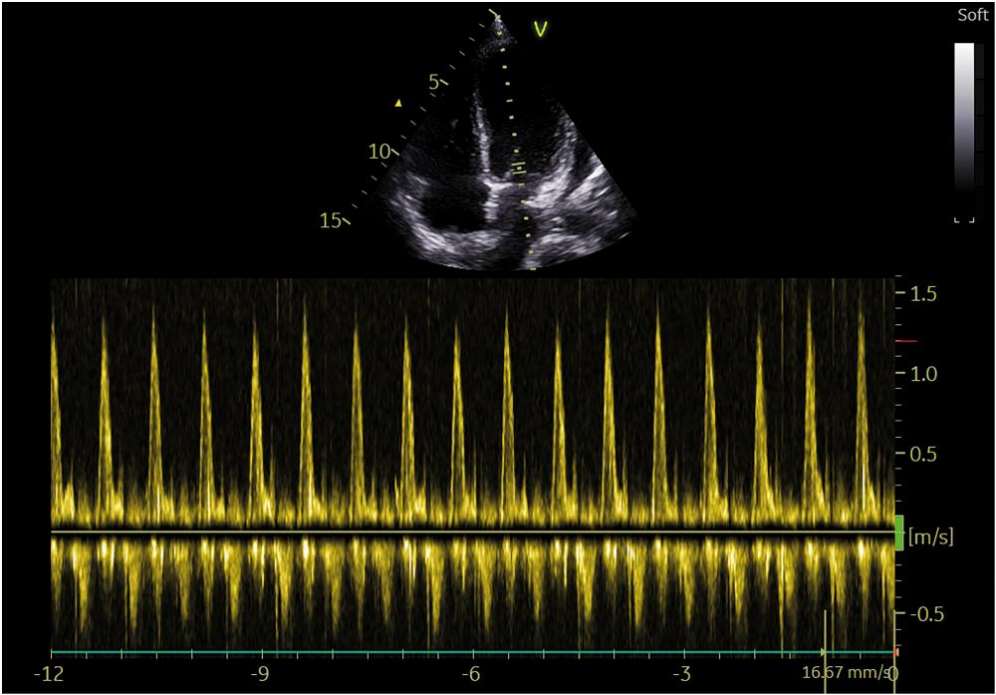
Transmitral pulse-wave Doppler. Transmitral pulse-wave Doppler showing no respiratory variability.

**Figure 4:**
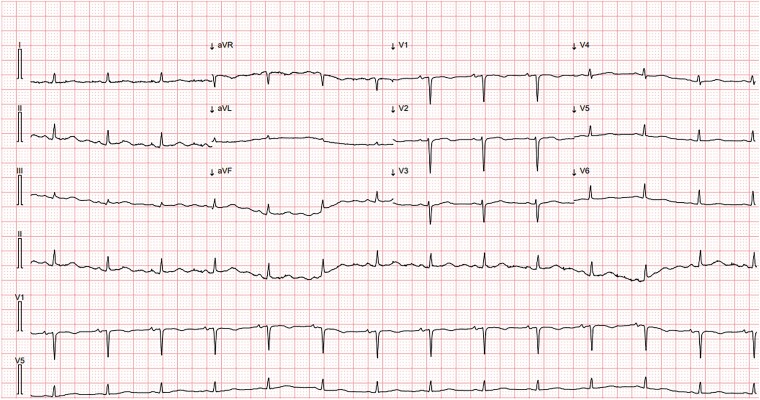
Electrocardiogram tracing showing low voltages due to pneumohydropericardium.

On Day 11, echocardiography revealed a pericardial effusion, more pronounced at the posterior level of the left ventricle (21 mm in parasternal long-axis) with the presence of air microbubbles inside, likely as a result of recent thoracic drainage removal, clearly visible also in apical four chambers (*Figures 1* and *2*; Supplementary material online, *Videos S1* and *S2*), without evidence of haemodynamic compromise: there was neither caval plethora nor evidence of respiratory variability of the transmitral Doppler (*Figure 3*). The electrocardiogram tracing revealed low voltage in the peripheral leads (*Figure 4*).

Since the pneumohydropericardium did not demonstrate a haemodynamic impact, it was monitored through serial echocardiographies: on Day 16, the air microbubbles were no longer visible on echocardiographic follow-up. The graft function was consistently preserved throughout the post-transplant period.

Also, on Day 17, the patient was discharged in good general and haemodynamic compensation. The patient was discharged with the following immunosuppressive therapy: mycophenolate mofetil 750 mg twice daily, tacrolimus 3 mg in the morning and 2.5 mg in the evening and prednisone 40 mg in the morning; the patient was not discharged with diuretics.

The patient was monitored during follow-up with weekly echocardiograms for the first month and biweekly echocardiograms for the second month, documenting the reduction and stabilization of the pericardial effusion at the 7-month follow-up (13 mm at the posterior level of the left ventricle in parasternal long-axis). The pericardial effusion has increased to 23 mm on echocardiography at the 9-month follow-up: the absence of air microbubbles persists, and the patient remains haemodynamically stable and continues to be monitored.

## Discussion

The pericardium is a double membrane that surrounds the heart and separates it from other thoracic structures. Normally, it contains a small amount of lubricating fluid that allows the heart to move smoothly during contractions.

Pneumohydropericardium is a medical condition characterized by the simultaneous presence of air and fluid in the pericardial space. This condition can result from various causes, including trauma, infections, invasive medical procedures such as pericardiocentesis, or complications arising from other cardiac or thoracic pathologies.^8^

The presence of air (pneumo-) and additional fluid (-hydropericardium) in this space can interfere with the normal functioning of the heart, potentially leading to complications such as compression of the cardiac chambers or cardiac tamponade, an urgent condition requiring immediate medical intervention.

The diagnosis of pneumohydropericardium is based on clinical observation, patient history, and the use of imaging techniques such as echocardiography and computerized tomography, which can reveal the presence of air and fluid in the pericardial space.

Management varies depending on the underlying cause and symptom severity. It may include conservative measures, such as monitoring and observation, or more aggressive interventions such as pericardiocentesis to remove air and fluid from the pericardial area, and treatments targeted at the underlying cause of the condition.^10^

In the literature, case reports of pneumohydropericardium with images similar to those presented in this article (thus with a clear interface between air bubbles and fluid) have been reported in only a few other cases, related mainly to tumours, and also to tuberculosis, chronic renal disease, and idiopatic.^1–7^ In these cases, the hydropericardium was treated through emergency pericardiocentesis^3,7^ or surgical drainage^2,4,5^ due to haemodynamic compromise or surgical closure of the fistulas that caused it.^6,8^ In our case, the treatment (conservative approach) differed as the patient had neither haemodynamic compromise nor any evident underlying causes that could be treated.

Around 20–35% of heart transplant recipients develop moderate to large pericardial effusion in the early postoperative period, with approximately half of these cases requiring drainage,^9,11^ but the occurrence of pneumohydropericardium has never been reported till now in this condition.

## Patient's perspective

The patient expressed deep concern when initially informed about the unusual presence of air bubbles around her heart following the transplant. Given her complex medical history and the challenges she faced during the transplant process, she feared additional complications and potential setbacks to her recovery. However, after thorough explanations provided by the medical team regarding the condition of pneumohydropericardium and its management through vigilant monitoring, the patient felt reassured. She understood that no invasive intervention was necessary and that her condition was under careful observation through serial echocardiographic assessments. The patient and her family appreciated the clear communication, and as her condition improved without further complications, she expressed gratitude for the care provided. She also valued the guidance on post-transplant monitoring and expressed a strong commitment to adhering to all medical follow-ups.

## Conclusion

This case underscores pneumohydropericardium as a complication that may exceptionally occur following heart transplantation. Through early recognition and consistent echocardiographic surveillance, we managed this rare condition conservatively in the acute phase. This case contributes to the sparse literature on pneumohydropericardium and underscores echocardiography as a critical tool in guiding management. We think that this case is clinically significant for transplant teams and cardiologists who may encounter such rare complications. Further investigation into similar cases may strengthen understanding and best practices, ensuring optimal outcomes for future heart transplant recipients facing this rare complication.

## Supplementary Material

ytaf062_Supplementary_Data

## Data Availability

The data that support the findings of this study are available from the corresponding author upon reasonable request.

## Lead author biography

Dr Filippo Brucato studied Medicine and Surgery at the University of Milan. He is currently a cardiology resident at first year of training at the University of Milano-Bicocca, presently based at Niguarda Hospital. His fields of interests are advanced heart failure and electrophysiology.

## Notes

**Consent:** The authors confirm that written consent for submission and publication of this case report including images and associated text has been obtained from the patient in line with the COPE guidance.

**Funding:** None declared.
